# Sirt6 deficiency aggravates angiotensin II-induced cholesterol accumulation and injury in podocytes

**DOI:** 10.7150/thno.45003

**Published:** 2020-06-12

**Authors:** Qian Yang, Jijia Hu, Yingjie Yang, Zhaowei Chen, Jun Feng, Zijing Zhu, Huiming Wang, Dingping Yang, Wei Liang, Guohua Ding

**Affiliations:** 1 Division of Nephrology, Renmin Hospital of Wuhan University, Wuhan, Hubei, China.; 2 Nephrology and Urology Research Institute of Wuhan University, Wuhan, Hubei, China.

**Keywords:** Sirt6, angiotensin II, cholesterol accumulation, podocyte injury, methyl-β-cyclodextrin

## Abstract

Disturbed renal lipid metabolism, especially cholesterol dysregulation plays a crucial role in the pathogenesis of chronic kidney disease (CKD). We recently reported that angiotensin (Ang) II could induce cholesterol accumulation and injury in podocytes. However, the underlying mechanisms for these alterations remain unknown.

**Methods:** Bioinformatics analysis of renal biopsy specimens from patients with hypertensive nephropathy (HN) suggests the involvement of Sirtuin 6 (Sirt6) in Ang II-induced dysregulation of glomerular cholesterol. Using a podocyte-specific Sirt6 knockout mouse model, the effects of Sirt6 on Ang II-induced cholesterol accumulation in podocytes and the therapeutic efficacies of cholesterol-lowering agents were evaluated.

**Results:** Cholesterol accumulation was detected in the podocytes of Ang II-infused mice, whereas selective deletion of Sirt6 in podocytes not only increased cholesterol accumulation in these cells but also exacerbated Ang II-induced kidney injury. Deletion of Sirt6 also attenuated the protective effect of cyclodextrin (CD) on Ang II-induced urinary albumin excretion, glomerulosclerosis and podocyte injury. In addition, we demonstrated that Sirt6 affected cholesterol efflux in podocytes by regulating the expression of ATP-binding cassette transporter G1 (ABCG1).

**Conclusions:** These findings provide evidence that Sirt6 is a potential target for renin-angiotensin system (RAS)-associated podocyte injury and provide a rationale for the application of cholesterol-lowering agents in patients with CKD.

## Introduction

Chronic kidney disease (CKD) is often accompanied by activation of the renin-angiotensin system (RAS) 1. Angiotensin (Ang) II, the main effector of RAS, has been shown to promote the progression of CKD, and its direct effect on podocyte injury has been extensively documented 2,3. The application of angiotensin-converting enzyme inhibitors (ACEIs) or angiotensin-receptor blockers (ARBs) cannot completely reverse podocyte injury or glomerulosclerosis 4,5, suggesting that other mechanisms are involved in Ang II-induced podocyte injury.

Emerging evidence suggests that cholesterol accumulation plays a vital role in podocyte injury induced by various factors, such as metabolism-related gene variation 6-8 and alteration in the extracellular environment 9-11. Recent studies demonstrated that apolipoprotein L1 (APOL1) mutation, which can lead to high-density lipoprotein (HDL) dysfunction and cholesterol efflux disorder, caused disordered intracellular endosome transport, enhanced the permeability of lysosomes and promoted podocyte necrosis 6-8. Forst *et al.* 9 reported that cholesterol mediates podocyte cytoskeletal rearrangement caused by mechanical stress. Several studies have demonstrated that promotion of cholesterol efflux with cyclodextrin (CD) or inhibition of cholesterol influx with LDL receptor (LDLR) knockdown significantly inhibited podocyte apoptosis under the condition of diabetic nephropathy 10-11.

We recently reported that Ang II could induce cholesterol accumulation and injury in podocytes 12. However, the underlying mechanisms remain unclear. In this study, we analyzed the microarray data of 14 patients with hypertensive nephropathy (HN) and 4 healthy controls, which revealed that the expression of a variety of cholesterol efflux-related genes was downregulated in renal biopsy specimens from the HN patients.

Sirtuin 6 (Sirt6) is a well-known nucleus‐associated deacetylase that belongs to the Sirtuin protein family 13. By deacetylating histones H3K9 and H3K56, Sirt6 participates in important biological activities such as cellular senescence 14, gene expression 15, genomic stability 16, and tumor suppression 17. Studies have shown that Sirt6 participates in the regulation of cellular lipid metabolism 18,19. Deletion of Sirt6 promoted the formation of fatty liver in mice and increased the cholesterol content in hepatocytes 20,21. Recent studies have demonstrated the protective role of Sirt6 in age-associated kidney dysfunction 22 and multiple podocyte diseases 23, but whether Sirt6 is involved in Ang II-induced podocyte injury and its role in podocyte cholesterol metabolism have not been reported. In the present study, we evaluated the effect of podocyte-specific Sirt6 deletion on cholesterol accumulation and injury in the podocytes of Ang II-infused mice. We also explored the possible pathways by which Sirt6 regulates cholesterol metabolism in Ang II-treated podocytes.

## Methods

### Microarray data collection and analysis

Genes involved in cholesterol metabolism were analyzed by microarray. The GSE37460 gene expression dataset was downloaded from the Gene Expression Omnibus (GEO, www.ncbi.nlm.nih.gov/geo/) database, which is a public database containing freely available microarray data. Glomerular specimens from patients with HN and healthy controls based on the GPL14663 platform were selected for analysis. Patients with biopsy-proven hypertensive nephrosclerosis (n=14) and healthy kidney transplant donors before kidney transplantation (n=4) from the European Renal cDNA Bank were included 24. The baseline characteristics of patients with nephrosclerosis and healthy controls with kidney biopsies are summarized in Table S1. None of the healthy controls had diabetes, hypertension, reduced kidney function, or signs of kidney damage. Patients with nephrosclerosis had a higher blood pressure and lower eGFR than the healthy controls. All raw data were downloaded from the GEO database.

The microarray data were quantile normalized using the limma package 25. Data preprocessing and differentially expressed gene (DEG) identification were performed using Bioconductor in R. Glomerular genes whose expression significantly differed between controls and HN patients were defined as follows and used for further analyses: upregulated genes exhibited a P value <0.05 and fold change >1.0 upon comparison, and downregulated genes exhibited a P value <0.05 and fold change >1.0 upon comparison. Twenty-four genes involved in the regulation of cholesterol homeostasis were selected for expression analysis. Fold change and false discovery rate data for the DEGs were extracted and are presented in an expression map. Expression data of Sirt6 and twenty-four genes involved in the regulation of cholesterol homeostasis were extracted to generate a volcano plot.

### Generation of podocyte-specific Sirt6-knockout mice

Hybrid floxed Sirt6 (Sirt6^flox/+^) mice on a C57BL/6J background were constructed by Cyagen Biotechnology (Guangzhou, China) and crossed to generate homozygous floxed Sirt6 (Sirt6^flox/flox^) mice. Nphs2-Cre transgenic mice (B6. Cg-Tg [NPHS2-Cre]) were obtained from the Jackson Laboratory (Bar Harbor, USA). Sirt6^flox/flox^ mice were crossed with Nphs2-Cre mice to generate podocyte-specific Sirt6-knockout (Sirt6^flox/flox^/Nphs2.Cre^+^) mice. Genotyping for podocyte-specific Sirt6-knockout mice was performed using the following primers: Cre- Forward: GCGGTCTGGCAGTAAAAACTATC; Cre- Reverse: GTGAAACAGCATTGCTGTCACTT; LoxP- Forward: GTCAGAATGACTCAATGTTGTGGA; LoxP- Reverse: TCCTGTGGTGGAGACAATGAC. Homozygous floxed mice without Cre expression (Sirt6^flox/flox^/Nphs2.Cre^-^ mice) were used as controls.

### Animal studies

All procedures complied with the Animal Management Rules of the Ministry of Health of the People's Republic of China and were approved by the Animal Care Committee of Wuhan University. An osmotic mini-pump (Alzet model 2004, CA) was embedded in each of the male Sirt6^flox/flox^/Nphs2.Cre^+^ and Sirt6^flox/flox^/Nphs2.Cre^-^ mice (8 weeks of age) used in this study, which were randomly assigned to receive a normal saline infusion or 700 ng/kg/min Ang II (Sigma-Aldrich, USA) infusion for 8 weeks (the mini-pump was replaced after 4 weeks.). The CD-treated animals were simultaneously given methyl-β-cyclodextrin (100 mg/kg/d, Sigma-Aldrich, USA) by subcutaneous injection. The simvastatin (SV)-fed groups were given SV (20mg/kg/d, Merck Sharp & Dohme, UK) by gavage. 24 h urine was collected from metabolic cages, and the albumin-to-creatinine ratio (ACR) was measured every two weeks. The animals were sacrificed, and their kidneys were perfused with physiological saline before isolation and storage at -80 °C for glomeruli isolation (sieve method), and biochemical and renal pathological analysis.

### Cell culture

Conditionally immortalized human podocytes kindly provided by Dr. Moin A. Saleem (Academic Renal Unit, Southmead Hospital, Bristol, UK) were cultured under standard conditions. The medium consisted of RPMI 1640 (HyClone, USA) containing 10% heat-inactivated fetal bovine serum (FBS; Gibco, USA), 100 U/mL penicillin G, 100 μg/mL streptomycin (Invitrogen, USA) and 1× insulin-transferrin-selenium (ITS; Invitrogen, USA) at 33 °C. To induce differentiation, podocytes were cultured at 37 °C for 10-14 d without ITS, and the differentiated podocytes were used in all experiments. The differentiated cells were stimulated with Ang II (10^-7^ M) for 24 h. For CD experiments, podocytes were pretreated with 5 mM methyl-β-cyclodextrin (Sigma-Aldrich, USA) for 1 h. For interference treatment, small interfering RNAs (siRNAs) targeting Sirt6 (Qiagen, Germany) were transfected into podocytes with HiPerFect (Qiagen, Germany) according to the manufacturer's instructions. Each experimental result was confirmed in three independent podocyte clones.

### Immunofluorescence assay

The frozen kidney sections were blocked and incubated with a mixture of guinea pig anti-adipocyte differentiation-related protein (Adrp) antibody (1:100, Progen Biotechnik, Germany) and mouse anti-WT1 antibody (1:100, Novus, USA) or a mixture of rabbit anti-Sirt6 antibody (1:100, Thermo Fisher Scientific, USA) and mouse anti-WT1 antibody (1:100, Novus, USA)/mouse anti-Synaptopodin antibody (1:100, Progen Biotechnik, Germany) overnight at 4 °C, followed by incubation with fluorescent secondary antibodies (1:200, Thermo Fisher Scientific, USA) at 37 °C for 90 min in the dark. Nuclei were counterstained with DAPI (Antgene, China) for 5 min. All microscopic images were recorded with fluorescence microscope (Olympus, Japan).

### Immunohistochemical assay

The paraffin-embedded kidney sections were deparaffinized, subjected to antigen retrieval and blocked. Antigen retrieval was performed in high-pressure citrate buffer (0.01 mol/L, pH 6.0) for 10 min, and the retrieved antigen was blocked with 10% goat serum for 30 min at room temperature. After incubation with 3% hydrogen peroxide for 10 min, the sections were incubated with anti-Sirt6 antibody (1:100, Thermo Fisher Scientific, USA) overnight at 4 °C. Then, the sections were incubated with polymerized horseradish peroxidase-conjugated secondary antibody for 30 min. After diaminobenzidine staining, the sections were counterstained with hematoxylin. Slides were viewed by microscopy (Olympus, Japan).

### Western immunoblotting

Total protein was extracted from isolated glomeruli and podocytes with RIPA buffer containing a protease inhibitor cocktail (P8340, Sigma-Aldrich). Equal amounts of protein were separated by SDS-PAGE and then transferred to PVDF membranes (Millipore Corp, USA). The membranes were incubated with a primary antibody (anti-ABCG1 rabbit polyclonal antibody, 1:500, Novus, USA; anti-ABCA1 mouse monoclonal antibody, 1:200, Abcam, UK; anti-HMGCR rabbit monoclonal antibody, 1:500, Abcam, UK; anti-α-Tubulin mouse monoclonal antibody, 1:1,000, Antgene, China) overnight at 4 °C. An Alex Fluor 680/790-labeled (1:10,000, LI-COR Biosciences, USA) goat anti-mouse/goat anti-rabbit antibody was used as the secondary antibody. Bands were visualized by a LI-COR Odyssey Infrared Imaging System.

### Apoptosis assays

The apoptosis of podocytes from kidney tissue was determined by transmission electron microscopy (TEM). Briefly, 1 mm^3^ kidney tissue sections that had been fixed in 2.5% glutaraldehyde were postfixed with 1% osmic acid, dehydrated in a graded series of ethanol solutions and embedded. The embedded tissues were sectioned, stained with uranyl acetate and lead citrate, and then observed and photographed with a Hitachi H-600 transmission electron microscope (Hitachi, Japan).

Apoptosis of podocytes from kidney tissue was determined by double immunofluorescence (IF) staining with WT-1 and TUNEL according to the manufacturer's instructions (Roche Applied Science, Germany).

Apoptosis in cultured podocytes was determined by flow cytometry using annexin V-FITC and 7-ADD double staining according to the manufacturer's instructions (FITC-Annexin V Apoptosis Detection Kit with 7-AAD, BioLegend, USA).

### Oil Red O staining

Kidney sections or cells on coverslips were fixed, rinsed with 60% isopropanol for 1 min, and stained with an Oil Red O (Sigma-Aldrich, USA) working solution for 30 min at room temperature. The slides were rinsed again for 1 min with 60% isopropanol and then returned to distilled water. Finally, the slides were counterstained with hematoxylin for 1 min. All slides were visualized using an Olympus camera (Japan).

### Filipin staining

The cholesterol content was analyzed with the cholesterol-binding compound filipin III (Sigma-Aldrich, USA). Kidney sections or cells on coverslips were fixed. Filipin staining (0.05 mg/mL filipin in a 1:4 solution of DMSO: PBS) was performed for 2 h at room temperature, and staining was analyzed by fluorescence microscopy (Olympus, Japan).

### Cholesterol quantification

The cholesterol content was determined using a cholesterol quantitation kit (Sigma-Aldrich, USA). Briefly, cells (1×10^6^) were extracted and centrifuged to remove insoluble material. Then, the organic phase was transferred to a new tube and air dried at 50 °C to remove any residual organic solvent. The dried lipids were dissolved in cholesterol assay buffer and then vortexed. Subsequently, the appropriate reaction mix was added and incubated for 60 min at 37 °C. Finally, the fluorescence intensity (λex=535 nm /λex=587 nm) was measured by a fluorescence microplate reader.

### Cholesterol efflux assay

Cholesterol efflux was determined using a cholesterol efflux assay kit (Sigma-Aldrich, USA). Differentiated human podocytes (1×10^5^ cells/well) were plated in a 96-well plate. After incubation for 2 h at 37 °C with 5% CO_2_, the cells were washed with RPMI 1640 medium (without FBS). Then, the appropriate reaction mix was added to label the cells. After being washed with RPMI 1640 medium (without FBS), the cells were incubated for 4 h with RPMI 1640 medium containing 10% FBS at 37 °C with 5% CO_2_. After incubation, the supernatant was kept, and the cell monolayer in each well was solubilized with 100 μL cell lysis buffer. Finally, the fluorescence (λex=482 nm /λex=515 nm) of the supernatant and the cell lysate was measured by a fluorescence microplate reader.

### Statistical analyses

All experiments were repeated at least 3 times. Quantitative data are presented as the mean ± SD, and statistical analyses were performed using SPSS v17.0. Statistical comparisons of groups were performed using one-way ANOVA, and the least-significant difference (LSD) test was used for multiple comparisons. Differences for which *P*<0.05 were considered statistically significant.

## Results

### Genes involved in cholesterol homeostasis are differentially expressed in the glomeruli of patients with HN compared with healthy controls

**Figure 1:**
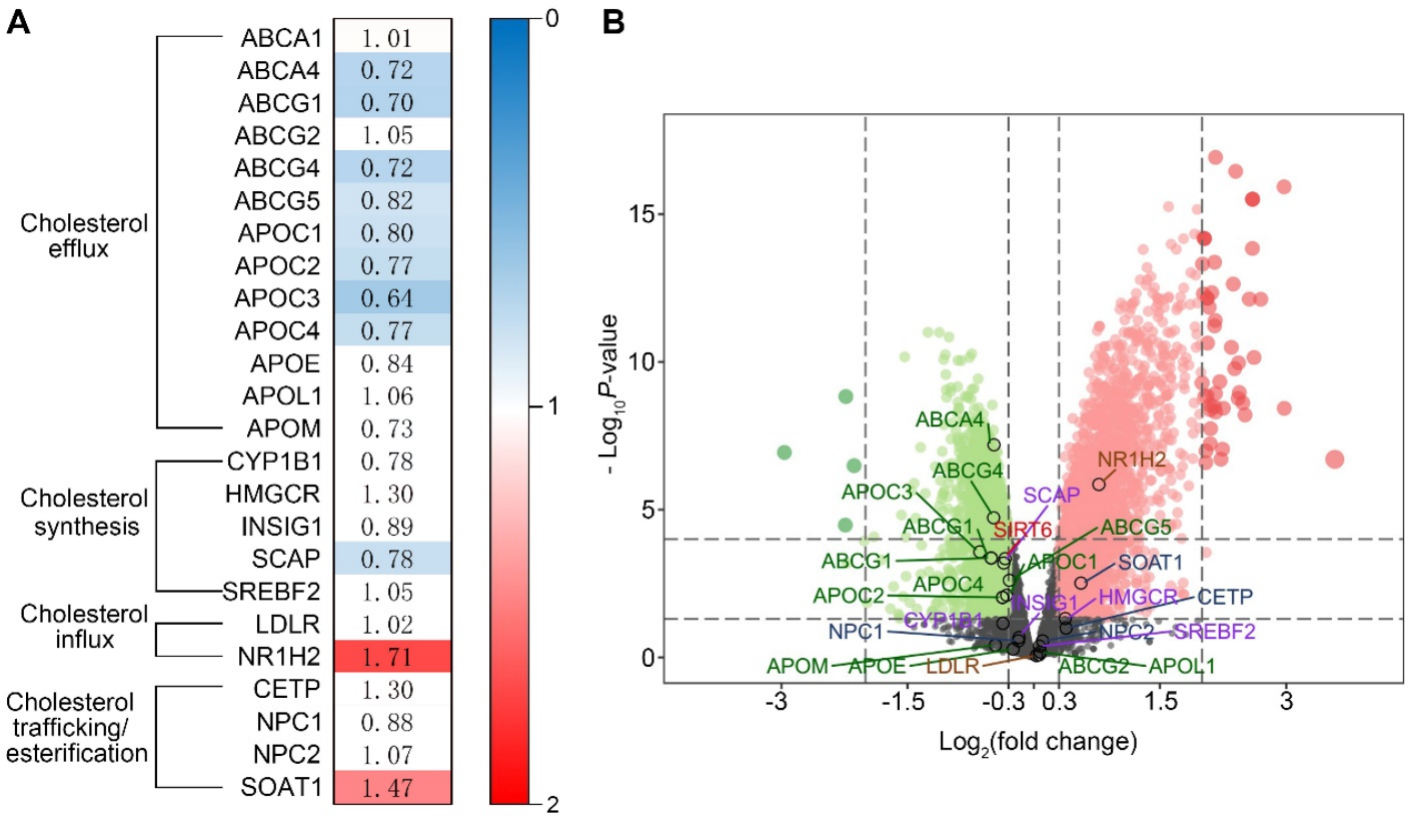
** Bioinformatics analysis of genes that regulate cholesterol homeostasis in patients with HN using the GSE37460 gene expression dataset.** (A) Fold change in glomerular gene expression in patients with HN compared with healthy controls. Blue blocks represent downregulated differentially expressed genes (DEGs), false discovery rate ≤0.05; red blocks represent upregulated DEGs, false discovery rate ≤0.05; white blocks represent DEGs with no statistical significance, false discovery rate ≥0.05. (B) Volcano plot of the DEGs (red and green indicate DEGs with a |log2 fold-change| >0.3 and false discovery rate <0.05. Black indicates no DEGs or DEGs with a false discovery rate ≥0.05).

To investigate whether cholesterol dysregulation is involved in the pathogenesis of HN, we evaluated the expression of genes involved in cholesterol homeostasis in patients with HN and healthy controls by bioinformatics analysis. We found that 11 cholesterol metabolism-related genes were differentially expressed in the glomeruli of HN patients compared with healthy controls. Among these genes, the expression of those that regulate intracellular cholesterol efflux was downregulated in the glomeruli of HN patients compared to healthy controls (Figure 1A), suggesting that a reduction of cholesterol efflux contributes to the pathogenesis of HN. To decipher the mechanism of cholesterol metabolic imbalance in HN, we investigated the expression of Sirt6, a gene that regulates cholesterol metabolism in hepatocytes 26 and foam cells 27 and is involved in Ang II-induced cardiomyocyte injury 28. As shown in Figure 1B, we found that a reduction in Sirt6 expression was associated with the downregulation of 8 genes that regulate cholesterol efflux (ABCA4, ABCG1, ABCG4, ABCG5, APOC1, APOC2, APOC3, and APOC4) (Figure 1B), suggesting that the downregulation of Sirt6 contributes to Ang II-induced cholesterol dysregulation.

### Selective deletion of Sirt6 in mouse podocytes

**Figure 2:**
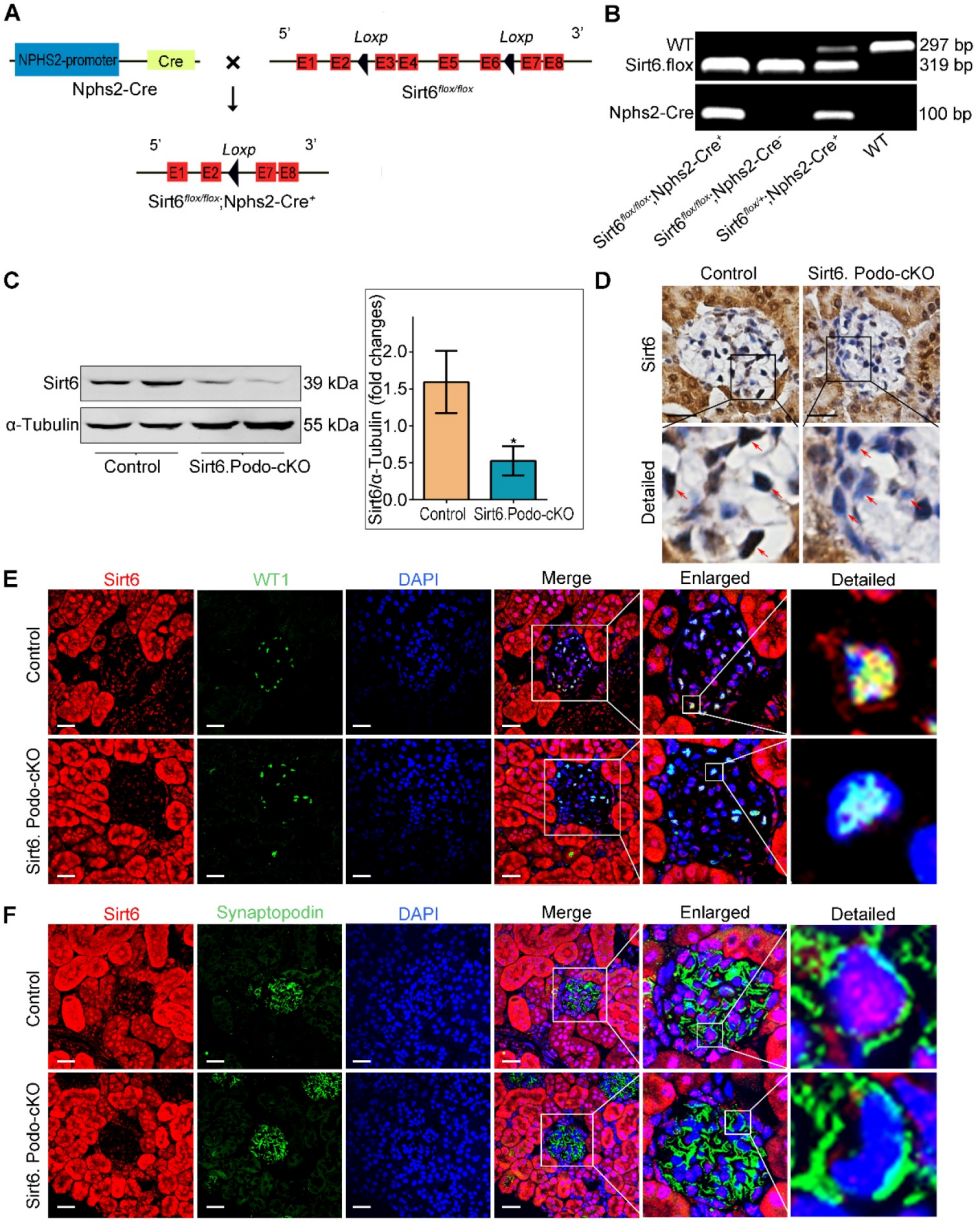
** Generation of podocyte-specific Sirt6 knockout mice.** (A) Schematic diagram of the construction of podocyte-specific Sirt6 knockout (Sirt6^flox/flox^/Nphs2.Cre^+^) mice. Exons 3-6 of Sirt6 were deleted in podocytes by the Cre-Loxp system. (B) Representative PCR image of tail genotyping of each group. (C) Representative Western blots of Sirt6 and quantification of Sirt6 protein levels in the glomeruli of each group. *P <0.05 versus Control, n=6. (D) Representative immunohistochemistry staining of glomerular Sirt6 in each group (arrows point to the regions of nuclear Sirt6 expression). Scale bars: 20 µm. (E and F) Podocyte-specific loss of Sirt6 was confirmed by immunofluorescent staining for Sirt6 in podocytes. WT1 and Synaptopodin were used as podocyte markers. Control: Sirt6^flox/flox^/Nphs2.Cre^-^ group; Sirt6.Podo-cKO: Sirt6 podocyte conditional knockout: Sirt6^flox/flox^/Nphs2.Cre^+^ group; WT: wild-type; WT1: Wilms' tumor-1. Scale bars: 20 µm.

We first examined the effect of Ang II on the expression of SIRT6 in glomeruli and podocytes *in vivo*. By Western blotting, we found that the expression of Sirt6 was significantly reduced in Ang II-infused mouse glomeruli compared to the glomeruli of saline-infused mice (Figure S1A). Moreover, immunofluorescence double staining for WT1 and Sirt6 showed that Ang II infusion significantly reduced the expression of Sirt6 in glomerular podocytes (Figure S1B). To explore the role of Sirt6 in Ang II-induced cholesterol metabolic imbalance in podocytes, we generated podocyte-specific Sirt6-knockout mice using the Cre-Loxp system (Figure 2A). Conditional knockout mice in which Sirt6 was specifically ablated in podocytes were identified by tail genotyping (Figure 2B). Deletion of Sirt6 in glomeruli from Sirt6^flox/flox^/Nphs2-Cre^+^ mice compared to that in glomeruli from Sirt6^ flox/flox^ /Nphs2-Cre^-^ mice were confirmed by Western blotting analysis (Figure 2C) and immunohistochemical staining (Figure 2D). The loss of Sirt6 in podocytes was verified by immunostaining (Figure 2E-F).

### Deletion of Sirt6 in podocytes aggravated Ang II-induced glomerular injury

**Figure 3:**
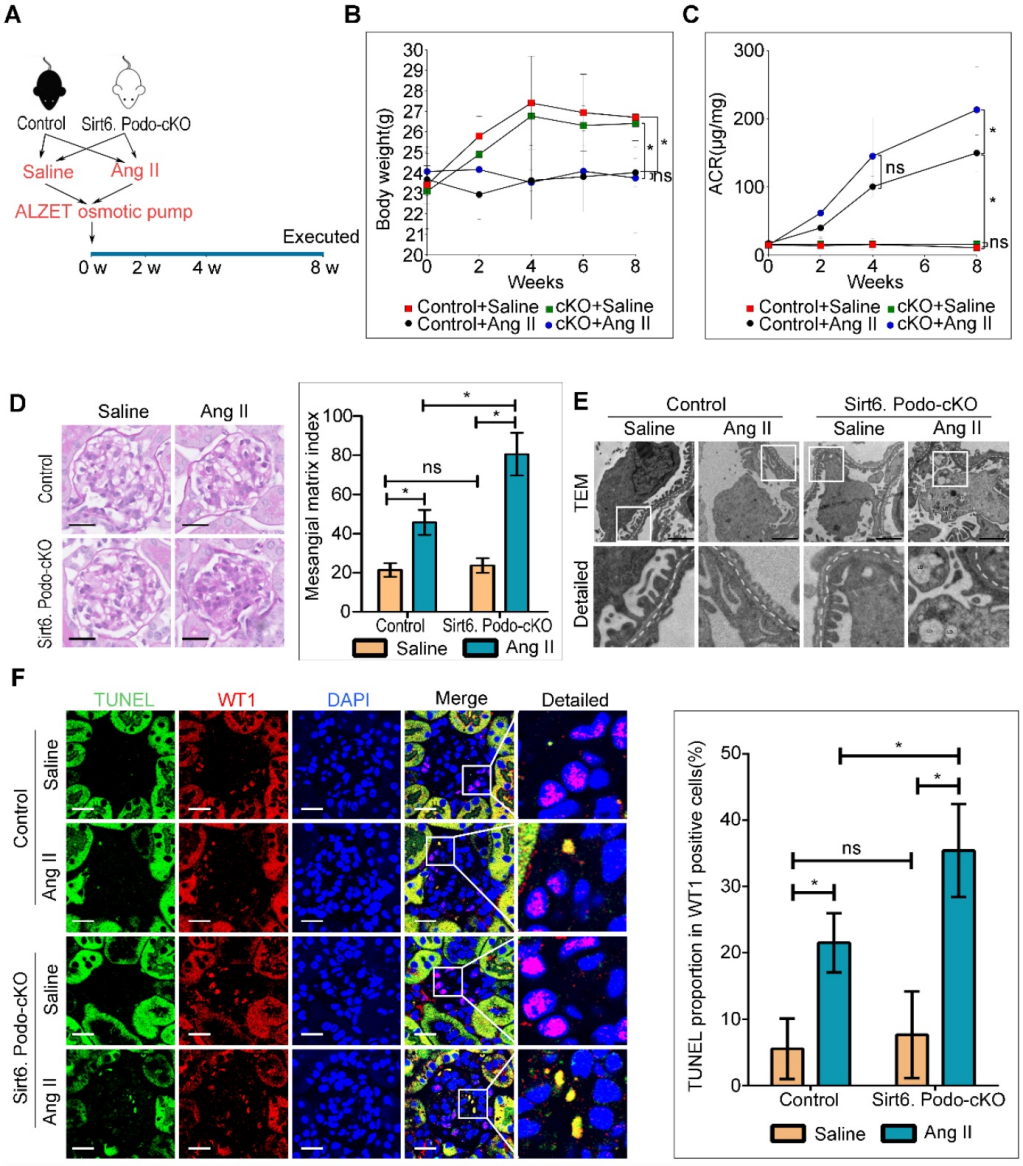
** Podocyte-specific deletion of Sirt6 aggravates Ang II-induced kidney injury (n=6).** (A) Schematic diagram of the construction of the Ang II-induced mouse model. (B) Quantitative analysis of body weight in the different groups. *P < 0.05. (C) Quantitative analysis of ACR (albumin-to-creatinine ratio) in the different groups. *P < 0.05. (D) Representative microscopy images and quantification of PAS staining of kidney sections for each group (original magnification ×400). Scale bars: 20 µm. (E) Representative transmission electron microscopy images of the ultrastructure of capillary loops in each group (original magnification×10,000). Scale bars: 2 µm. (F) Representative microscopy images of WT1 and TUNEL double staining of kidney sections for each group (original magnification×600) and quantification of apoptotic podocytes. Scale bars: 20 µm. *P < 0.05. Control: Sirt6^flox/flox^/Nphs2.Cre^-^ group; Sirt6.Podo-cKO=cKO: Sirt6 podocyte conditional knockout: Sirt6^flox/flox^/Nphs2.Cre^+^ group; LDs: lipid droplets; WT1: Wilms' tumor-1.

To explore the role of Sirt6 in Ang II-induced podocyte injury, we induced kidney injury with Ang II in podocyte-specific Sirt6 knockout and wild type mice (Figure 3A). Both Sirt6^flox/flox^/Nphs2-Cre^+^ and Sirt6^ flox/flox^/Nphs2-Cre^-^ mice infused with Ang II displayed weight loss similar in extent to that of saline-infused control mice (Figure 3B). However, we observed a significant increase in urinary albumin excretion after 8 weeks of infusion in Ang II-infused Sirt6^flox/flox^/Nphs2-Cre^+^ mice compared to Ang II-infused Sirt6 ^flox/flox^/Nphs2-Cre^-^ mice (Figure 3C). Periodic acid Schiff (PAS) staining revealed a marked increase in mesangial expansion and glomerulosclerosis in Ang II-infused Sirt6^flox/flox^/Nphs2-Cre^+^ mice compared with Ang II-infused Sirt6^flox/flox^/Nphs2-Cre^-^ controls mice (Figure 3D). Consistent with the increase in glomerular injury in Ang II-infused Sirt6^flox/flox^/Nphs2-Cre^+^ mice, we detected exacerbated effacement of the foot process (Figure 3E and Figure S2A) and increased apoptosis in podocytes from Ang II-infused Sirt6^flox/flox^/Nphs2-Cre^+^ mice (Figure 3F). Previous studies demonstrated that a reduction in WT1-positive podocytes plays an important role in the development of proteinuria and renal disease progression 29, 30. We evaluated the effect of Sirt6 deficiency on the number of WT-1-positive cells. As shown in Figure S2B, the number of WT-1-positive cells was decreased in the glomeruli from Ang II-infused mice compared with those from saline-infused mice, and this number was further reduced by Sirt6 deletion. These results suggest that Sirt6 deficiency aggravates Ang II-induced glomerular podocyte loss. Interestingly, a large number of lipid droplets (LDs) were observed in podocytes from Ang II-infused Sirt6^flox/flox^/Nphs2-Cre^+^ mice (Figure 3E), providing direct evidence of the involvement of Sirt6 in podocyte lipid metabolism.

### Deletion of Sirt6 aggravated Ang II-induced cholesterol accumulation in podocytes

**Figure 4:**
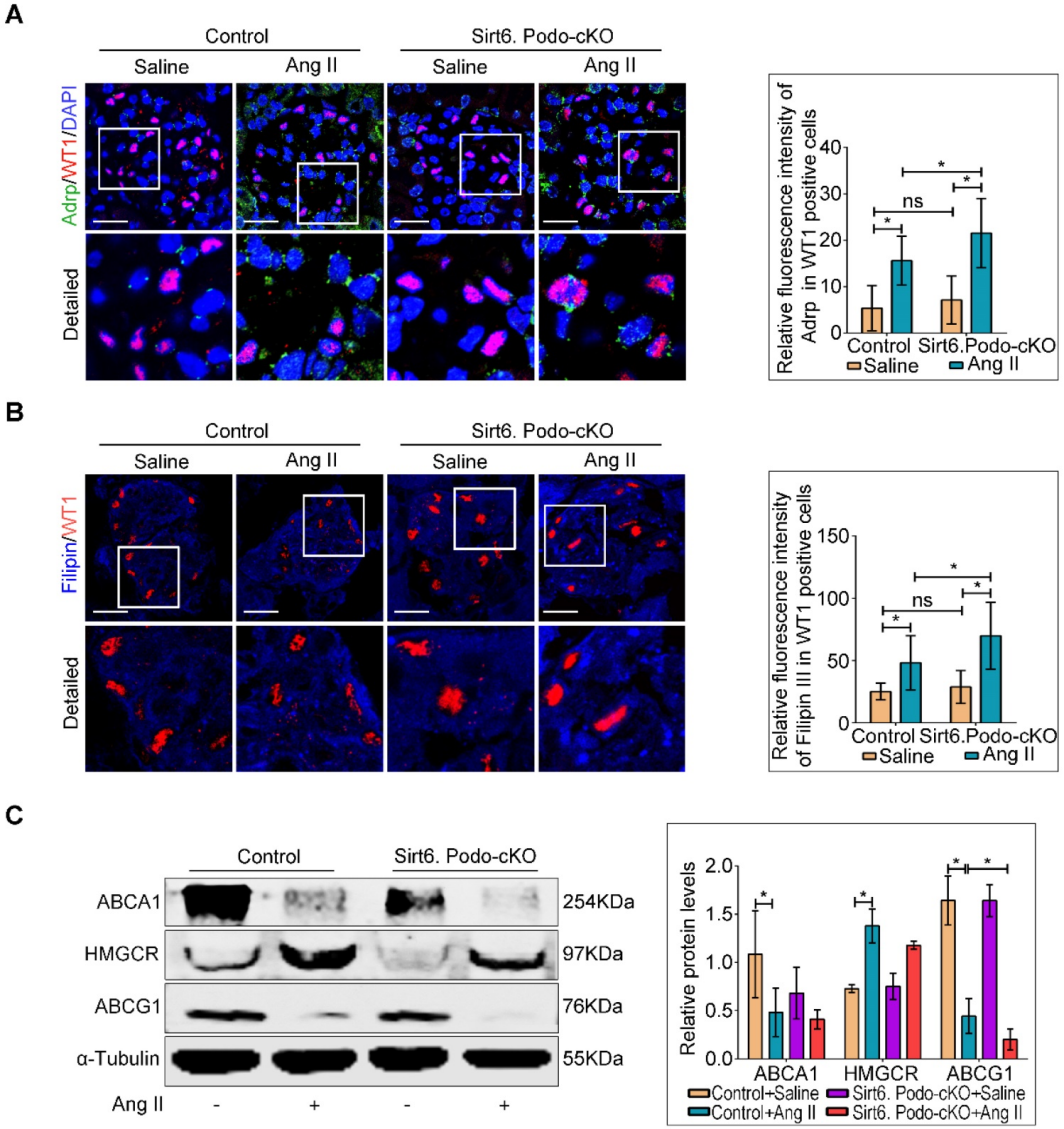
** Podocyte-specific deletion of Sirt6 aggravates Ang II-induced cholesterol accumulation in podocytes *in vivo*.** (A) Representative microscopy images and quantification of WT1 and Adrp double staining of kidney sections for each group (original magnification×600). *P < 0.05, n=30. Scale bars: 20 µm. (B) Representative microscopy images and quantification of filipin and WT1 double staining of kidney sections in each group (original magnification ×600). *P < 0.05, n=30. Scale bars: 20 µm. (C) Representative Western blots and quantification of ABCA1, HMGCR and ABCG1 protein levels in each group. *P < 0.05, n=3. ABCA1: ATP-binding cassette transporter A1; ABCG1: ATP-binding cassette transporter G1; Adrp: adipocyte differentiation-related protein; HMGCR: 3-hydroxy-3-methylglutaryl coenzyme A reductase; LDLR: low-density lipoprotein receptor; Control: Sirt6^flox/flox^/Nphs2.Cre^-^ group; Sirt6.Podo-cKO: Sirt6 podocyte conditional knockout: Sirt6^flox/flox^/Nphs2.Cre^+^group; WT1: Wilms' tumor-1.

Although Sirt6 is involved in the regulation of cholesterol metabolism in hepatocytes 26 and foam cells 27, its role in cholesterol homeostasis in podocytes is unknown. To evaluate whether the loss of Sirt6 affects Ang II-induced cholesterol accumulation in podocytes, Oil Red O staining (Figure S3) and immunofluorescence staining for Adrp (Figure 4A) were performed to detect the LDs. We found that deletion of Sirt6 promoted Ang II-induced LDs formation in podocytes (Figure 4A and Figure S3). Second, we evaluated the cholesterol content in podocytes by filipin staining. We found that the cholesterol content was significantly increased in the podocytes of the Ang II-infused Sirt6^flox/flox^/Nphs2-Cre^+^ mice compared to that in podocytes of the Ang II-infused Sirt6^flox/flox^/Nphs2-Cre^-^ mice (Figure 4B). We next analyzed the glomerular expression of several important molecules associated with cholesterol homeostasis by Western blot analysis. The expression of ABCG1 was significantly reduced in Ang II-infused Sirt6^flox/flox^/Nphs2-Cre^+^ mouse glomeruli compared to Ang II-infused Sirt6^flox/flox^/Nphs2.Cre^-^ mouse glomeruli, and the deletion of Sirt6 had no obvious effect on the expression of ABCA1 or HMGCR in these mice (Figure 4C). These results suggest that Sirt6 regulates Ang II-induced podocyte injury by influencing ABCG1-mediated cholesterol efflux.

### Deletion of Sirt6 attenuated the protective effects of CD against Ang II-induced podocyte injury *in vivo*

**Figure 5:**
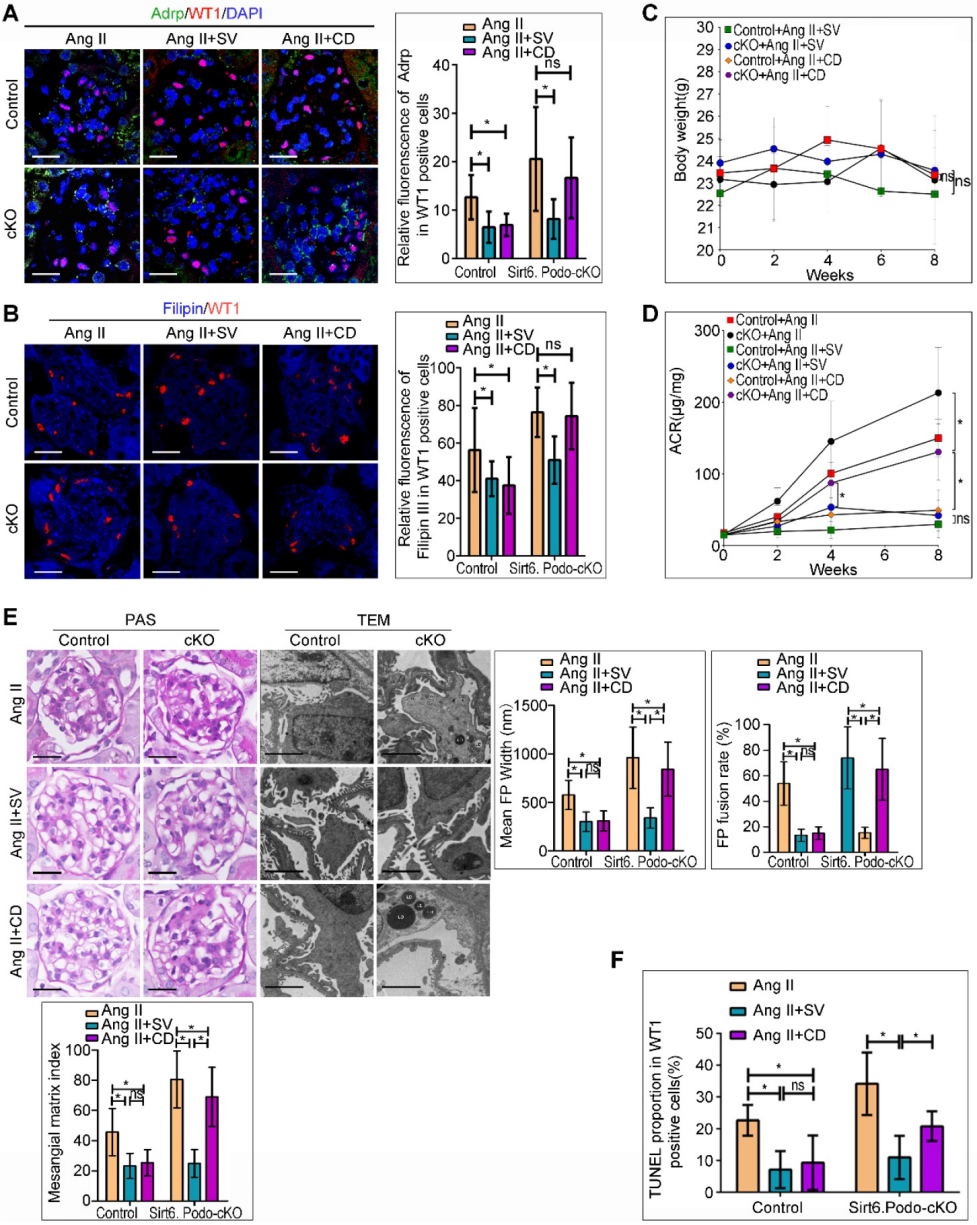
** Podocyte-specific deletion of Sirt6 affects the protective effect of cholesterol-lowering agents on Ang II-induced injury in podocytes.** (A) Representative microscopy images and quantification of WT1 and Adrp double staining of kidney sections for each group (original magnification×600). *P < 0.05, n=30. Scale bars: 20 µm. (B) Representative microscopy images and quantification of filipin and WT1 double staining of kidney sections in each group (original magnification×600). *P < 0.05, n=30. Scale bars: 20 µm. (C) Quantitative analysis of body weight in different groups. *P < 0.05, n=6. (D) Quantitative analysis of ACR (albumin-to-creatinine ratio) in different groups. *P < 0.05, n=6. (E) Representative microscopy images and quantification of PAS of kidney sections for each group (original magnification ×400), Scale bars: 20 µm; Representative transmission electron microscopic images of the ultrastructure of capillary loops (original magnification×10,000) and quantitation of foot process effacement in each group, Scale bars: 2 µm. (F) Quantification of apoptotic podocytes of WT1 and TUNEL double staining of kidney sections for each group (original magnification×600). *P < 0.05, n=6. Control: Sirt6^flox/flox^/Nphs2.Cre^-^ group; cKO: Sirt6 podocyte conditional knockout: Sirt6^flox/flox^/Nphs2.Cre^+^ group; Ang II: Ang II-infused group; Ang II+SV: Ang II-infused and simvastatin administration group; Ang II+CD: Ang II-infused and CD administration group; CD: cyclodextrin; LDs: lipid droplets; TEM: transmission electron microscopy; WT1: Wilms' tumor-1.

Treatment with SV and CD, the cholesterol-lowering agents which inhibits cholesterol synthesis and promotes cholesterol efflux, respectively, protects podocytes from injury caused by cholesterol accumulation 10,31-34. We evaluated whether deletion of Sirt6 affects the efficacy of SV and CD on podocyte injury and cholesterol metabolism in Ang II-induced mice *in vivo* (Figure S4A). As shown in Figure 5A and 5B, deletion of Sirt6 did not affect the protective role of SV in reducing Ang II-induced podocyte cholesterol accumulation, whereas the cholesterol-lowering effect of CD was abrogated in glomerular podocytes from Sirt6^flox/flox^/Nphs2-Cre^+^ mice compared to Sirt6 ^flox/flox^ /Nphs2-Cre^-^ mice infused with Ang II. Loss of Sirt6 had no effect on body weight loss in agent-treated mice (Figure 5C). In addition, we found that deletion of Sirt6 did not affect the protective effect of SV on Ang II-induced urinary albumin excretion (Figure 5D), glomerulosclerosis (Figure 5E) or podocyte injury (Figure 5E, 5F and Figure S4B). In contrast, deletion of Sirt6 had significant negative effects on CD-exerted protection in podocytes, as evidenced by the ACR, glomerulosclerosis, foot processes fusion and apoptosis rates of podocytes in Sirt6^flox/flox^/Nphs2-Cre^+^ mice treated with CD, which remained high (Figure 5D-5F and Figure S4B), in contrast, the above indicators in the other three treatment groups were essentially normal (Figure 5D-5F). These results suggest that Sirt6 may play a role in the regulation of Ang II-induced cholesterol accumulation and injury in podocytes by influencing cholesterol efflux.

### Sirt6 knockdown attenuated the protective effect of CD against Ang II-induced podocyte injury *in vitro*

**Figure 6:**
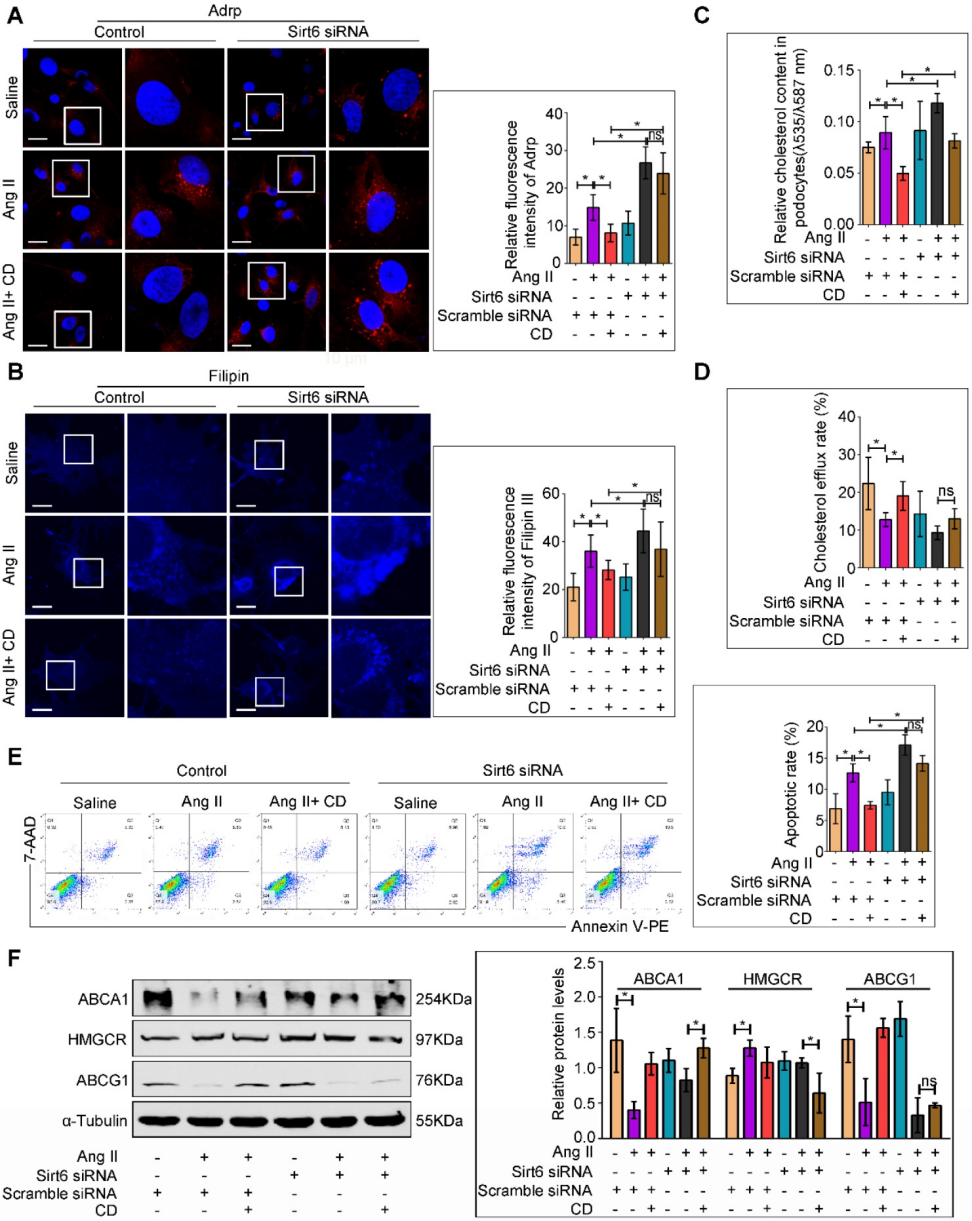
** Deletion of Sirt6 affects the protective effect of CD on Ang II-induced podocyte injury *in vitro*.** Podocytes were transfected with scrambled siRNA, or Sirt6 siRNA before pretreatment with 5 mM CD for 1 h and then stimulated with 10^-7^ M Ang II for 24 h. (A) Representative microscopy images and quantification of Adrp staining in each group (original magnification×600). *P < 0.05, n=20. Scale bars: 10 µm. (B) Representative microscopy images and quantification of filipin staining in each group (original magnification×600). *P < 0.05, n=20. Scale bars: 10 µm. (C) Quantitative analysis of cholesterol content in each group. *P < 0.05, n=9. (D) Quantitative analysis of cholesterol efflux rate in each group. *P < 0.05, n=6. (E) Flow cytometry analysis of the apoptotic rate of podocytes in each group. *P < 0.05, n=4. (F) Representative Western blots and quantification of ABCA1, HMGCR and ABCG1 protein levels in each group. *P < 0.05, n=3. ABCA1: ATP-binding cassette transporter A1; ABCG1: ATP-binding cassette transporter G1; Adrp: adipocyte differentiation-related protein; CD: cyclodextrin; HMGCR: 3-hydroxy-3-methylglutaryl coenzyme A reductase; LDLR: low-density lipoprotein receptor.

We further investigated whether Sirt6 deletion could attenuated the protective effects of CD on Ang II-induced podocyte injury *in vitro*. Oil Red O staining and immunofluorescence staining for Adrp showed that CD prevented LDs formation in Ang II-treated podocytes, and Sirt6 siRNA pretreatment abolished its improvement of lipid accumulation (Figure S5 and 6A). Moreover, CD treatment reduced Ang II-induced cholesterol accumulation in podocytes, and Sirt6 silencing by siRNA interference weakened the CD-induced reduction in cholesterol, as evidenced by filipin staining and cholesterol quantification (Figure 6B and 6C). In addition, cholesterol efflux analysis showed that CD-induced cholesterol efflux was partially eliminated by knockdown of Sirt6 (Figure 6D). Consistent with the results of cholesterol analysis, CD treatment significantly protected podocytes from Ang II-induced apoptosis, and knockdown of Sirt6 partially counteracted the protective effect of CD (Figure 6E). To explore the possible molecular mechanisms, we further analyzed the expression of cholesterol efflux-related proteins in each group. As shown in Figure 6F, treatment with Ang II decreased the expression of ABCA1 and ABCG1 in podocytes, which could be partially reversed by cotreatment with CD, whereas the effect of CD on ABCG1 expression in Ang II-treated podocytes was eliminated by knockdown of Sirt6. These results suggest that the protective effects of CD in podocytes are Sirt6 dependent and that Sirt6 affects cholesterol efflux in podocytes by regulating the expression of ABCG1.

### Sirt6 overexpression ameliorated Ang II-induced cholesterol accumulation and injury in podocytes *in vitro*

**Figure 7:**
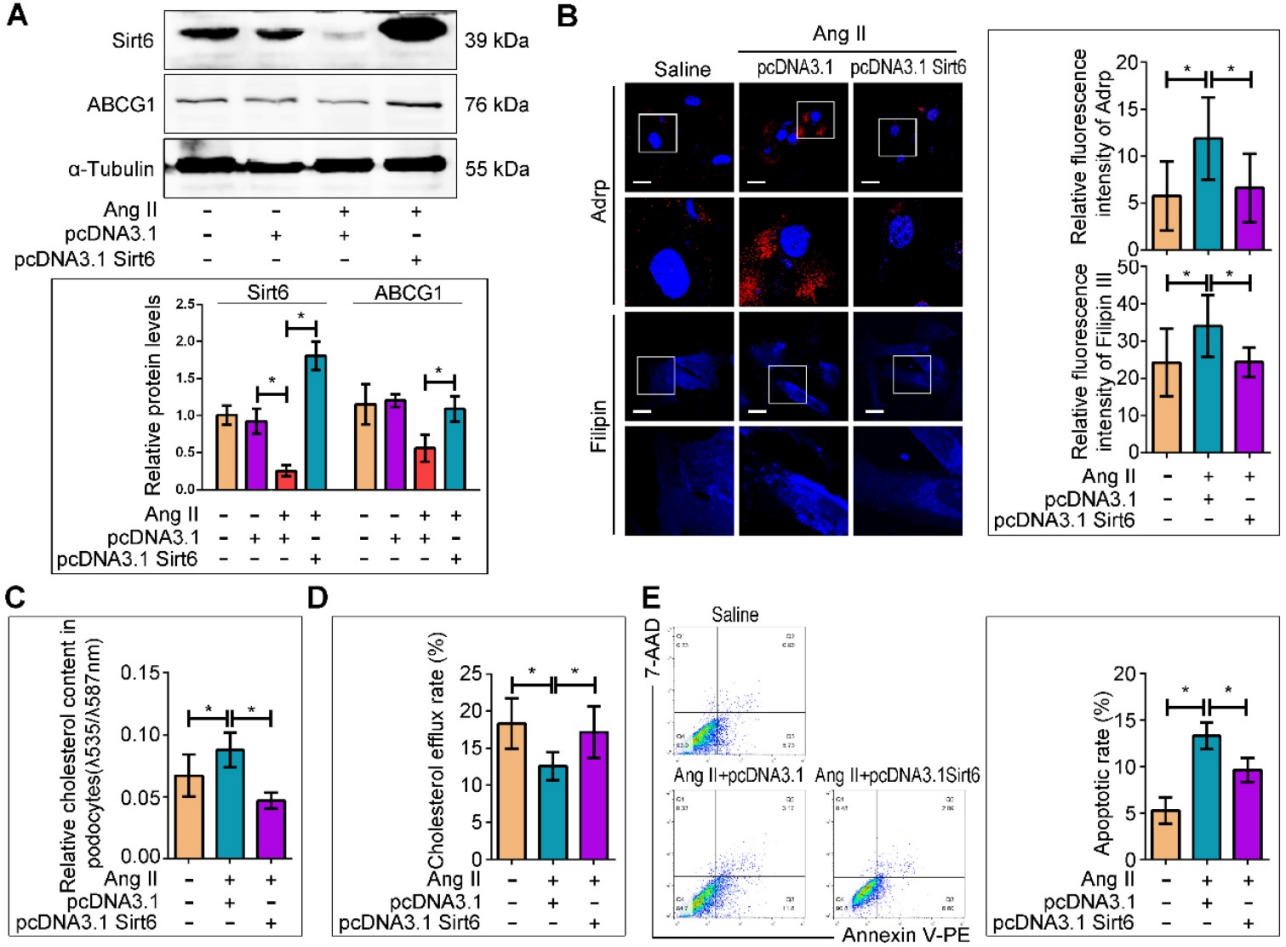
** Sirt6 overexpression interfered Ang II-induced cholesterol accumulation and injury in podocytes *in vitro*.** The podocytes were transfected with no plasmid, pcDNA3.1, or pcDNA3.1-Sirt6 and then stimulated with 10^-7^ M Ang II for 24 h. (A) Representative Western blots of Sirt6/ABCG1 protein levels in each group, *P < 0.05, n=3. (B) Representative microscopy images and quantification of Adrp and filipin staining in each group (original magnification×600). *P < 0.05, n=20. Scale bars: 10 µm. (C) Quantitative analysis of cholesterol content in each group. *P < 0.05, n=8. (D) Quantitative analysis of cholesterol efflux rate in each group. *P < 0.05, n=12. (E) Flow cytometry analysis of the apoptotic rate of podocytes in each group. *P < 0.05, n=4. ABCG1: ATP-binding cassette transporter G1.

We next evaluated whether Sirt6 overexpression could prevent Ang II-induced cholesterol accumulation and injury in podocytes. A recombinant plasmid (pcDNA3.1-Sirt6) was transfected into cultured podocytes to overexpress Sirt6. The efficiency of plasmid transfection was confirmed by Western blot analysis, and we found that Sirt6 expression was elevated in the plasmid-transfected cells under Ang II stimulation (Figure 7A). Overexpression of Sirt6 significantly ameliorated Ang II-induced LDs formation, cholesterol accumulation, and cholesterol efflux destruction (Figure 7B-7D) and restored ABCG1 expression (Figure 7A). In addition, overexpression of Sirt6 protected podocytes from Ang II-induced apoptosis (Figure 7E).

## Discussion

Accumulated evidence has indicated that renal lipid metabolic disorders contribute to the progression of CKD 10,35-36. Several recent studies suggested that lipid metabolites could serve as biomarkers in the early diagnosis and prognostic judgment of CKD. Profound changes in lipid metabolites in patients with CKD were detected by lipidomic analysis 37, and some lipid metabolites, such as lysophosphatidylethanolamine (20:0) and cholic acid, were identified as biomarkers for the CKD progression 38. Furthermore, lipid species, including chenodeoxycholic acid (CDCA) and glucosylceramide, were found to contribute to the discrimination of CKD patients with microalbuminuria and macroalbuminuria 39. Various investigation has demonstrated that intracellular cholesterol accumulation is strongly associated with podocyte damage in metabolic 40,41, nonmetabolic 33 and inherited diseases 42. Ang II, the main active molecule of the RAS system, has been shown to play a role in the initiation and progression of CKD 5, but its role in the cholesterol metabolism of podocytes remains unclear. A previous study assessed Ang II level in the renal biopsy specimens from HN patients and documented a significant increase, suggesting that Ang II plays an important role in the pathogenesis and progression of HN 43. In this study, our bioinformatics analysis of renal biopsy specimens from patients with HN provides insight into the involvement of Ang II in regulating cholesterol metabolism in the kidney and the participation of Sirt6 in Ang II-induced cholesterol dysregulation. To explore the role of Sirt6 in Ang II-induced cholesterol metabolic imbalance in podocytes, we evaluated the effects of podocyte-specific Sirt6 deletion on not only Ang II-induced kidney injury but also the therapeutic efficacy of cholesterol-lowering agents in protecting against podocyte injury. We demonstrated for the first time that cholesterol accumulation occurred in the podocytes of Ang II-infused mice, and that the selective deletion of Sirt6 in podocytes exacerbated Ang II-induced kidney injury and cholesterol accumulation in podocytes. Moreover, the deletion of Sirt6 did not affect the protective effect of SV against Ang II-induced increases in urinary albumin excretion, glomerulosclerosis and podocyte injury. In contrast, the podocyte-protective effects of CD (a cholesterol efflux promoter) were interfered by Sirt6 deletion. In addition, these findings were further confirmed by corresponding *in vitro* experiments. These results suggest that Sirt6 is involved in the regulation of Ang II-induced cholesterol accumulation and injury in podocytes by influencing cholesterol efflux. We further analyzed the expression levels of cholesterol efflux-related proteins to explore the possible mechanism of these effects; our results suggest that the protective effects of CD in podocytes are Sirt6 dependent and that Sirt6 may affects cholesterol efflux in podocytes by regulating the expression of ABCG1.

Sirt6 is an important member of the Sirtuin family that has been shown to deacetylate histones H3K9 and H3K56, participating in the regulation of cholesterol metabolism in hepatocytes and foam cells 21,27. Further studies on the underlying mechanisms revealed that Sirt6 can directly bind SREBP2, regulate its activity and influence the expression of downstream genes involved in the biosynthesis and uptake of cholesterol 44. A recent study showed that Sirt6 reduced the formation of macrophage foam cells by inducing cholesterol efflux under oxidized low-density lipoprotein (ox-LDL) conditions 27. These findings suggest that Sirt6 participates in the regulation of cholesterol metabolism by influencing the uptake, synthesis or efflux of cholesterol. The results of these studies support our hypothesis that the selective deletion of Sirt6 in podocytes exacerbates Ang II-induced cholesterol accumulation in podocytes. The present experiments confirmed our hypothesis and demonstrated that the deletion of Sirt6 further aggravated the downregulation of ABCG1 expression in Ang II-infused glomeruli, but had no obvious influence on the expression of ABCA1 or HMGCR. These results suggest that Sirt6 regulates Ang II-induced podocyte injury by influencing ABCG1-mediated cholesterol efflux.

ATP-binding cassette transporter G1 (ABCG1) is an integral membrane protein belonging to the ABC transporter family that regulates cellular cholesterol homeostasis 45 ABCG1 moves excess cholesterol from cells to HDL particles and initiates the process of reverse cholesterol transport 46,47. Wang *et al.* 48 reported that LXR activation induced the redistribution of ABCG1 from intracellular sites to the plasma membrane and increased cholesterol mass efflux to CD in an ABCG1-dependent fashion in macrophages. These findings may explain why the Sirt6-mediated downregulation of ABCG1 expression eliminates CD-induced cholesterol efflux in podocytes. Furthermore, all these findings indicate that the protective effects of CD in podocytes are Sirt6 dependent.

Previous studies indicated that Sirt6 can affect the expression of ABCG1 through multiple pathways. H3K9 could directly bind to the ABCG1 promoter and regulate the expression of ABCG1 49,50, and SIRT6 was demonstrated to deacetylate H3K9 51, suggesting that the Sirt6/H3K9 pathway is involved in the regulation of ABCG1 expression. Recent studies have demonstrated that the Sirt6/miR33 pathway is involved in the regulation of ABCG1 expression 27,52. Notch activation plays an important role in podocyte injury induced by several stimuli, including Ang II 53, 54, high glucose 23 and adriamycin 23. Moreover, Notch activation was associated with the downregulation of ABCG1 in the livers of ob/ob obese mice 55. Furthermore, blockade of Notch signaling with a neutralizing antibody against the Notch ligand Delta-like 4 (Dll4) resulted in decreased cholesterol accumulation in the livers of Ldlr^-/-^ mice 56. These studies suggest that Notch signaling may be another possible pathway through which ABCG1 expression is regulated by Sirt6. However, additional studies are needed to determine the exact pathway by which Sirt6 affects ABCG1 expression in podocytes in response to Ang II.

In conclusion, our study demonstrates for the first time that deletion of Sirt6 in podocytes exacerbates Ang II-induced kidney injury and cholesterol accumulation in podocytes, and that Sirt6 may regulate Ang II-induced podocyte injury by influencing ABCG1-mediated cholesterol efflux. These findings provide evidence that Sirt6 may be a potential target for RAS-associated podocyte injury and provide a rationale for the application of cholesterol-lowering agents in patients with CKD.

## Supplementary Material

Supplementary figures and tables.

## Abbreviations

ABCA1: ATP-binding cassette transporter A1
ABCG1: ATP-binding cassette transporter G1
ACEIs: angiotensin-converting enzyme inhibitors
ACR: albumin-to-creatinine ratio
Adrp: adipocyte differentiation-related protein
APOL1: apolipoprotein L1
ARBs: angiotensin-receptor blockers
CD: cyclodextrin
CDCA: chenodeoxycholic acid
CKD: diabetic kidney disease
DEG: differentially expressed gene
Dll4: Delta-like 4
FBS: fetal bovine serum
GEO: Gene Expression Omnibus
H3K9: Histone H3 lysine 9
H3K56: Histone H3 lysine 56
HDL: high-density lipoprotein
HMGCR: 3-hydroxy-3-methylglutaryl coenzyme A reductase
HN: hypertensive nephropathy
ITS: insulin-transferrin-selenium
LDs: lipid droplets
LDLR: low-density lipoprotein receptor
LXR: liver X receptor
NPHS2: Nephrosis2
Sirt6: Sirtuin 6
RAS: renin-angiotensin system
siRNAs: small interfering RNAs
SV: simvastatin
TEM: transmission electron microscopic
WT: wild type
WT1: Wilms' tumor-1
