# Supplementary material for: Sirt6 deficiency aggravates angiotensin II-induced cholesterol accumulation and injury in podocytes
Source: Theranostics. 2020 Jun 12;10(16):7465–79. doi: 10.7150/thno.45003 (PMC7330847; doi:10.7150/thno.45003)
Supplement: Supplementary file 1 — Supplementary figures and tables. [file thnov10p7465s1.pdf]

Table S1. Baseline characteristics of participants

	Healthy controls	Hypertensive nephrosclerosis
Age (y)	47.2±2.4	57.1±4.7
Male gender (%)	50	78.6
Systolic BP (mmHg)	< 140	146±22.9
Diastolic BP (mmHg)	< 80	88±13.5
Cholesterol (mmol/L)	absent	6.3±1.0
DM (%)	0	0
eGFR (ml/min per 1.73 m^2)	105.4±30.9	40.9±23.8
ACR (mg/mmol)	< 3.0	57±53
Therapy	absent	absent

BP, blood pressure; DM, diabetes mellitus; eGFR, estimated glomerular filtration rate; ACR, albumin-to-creatinine ratio.

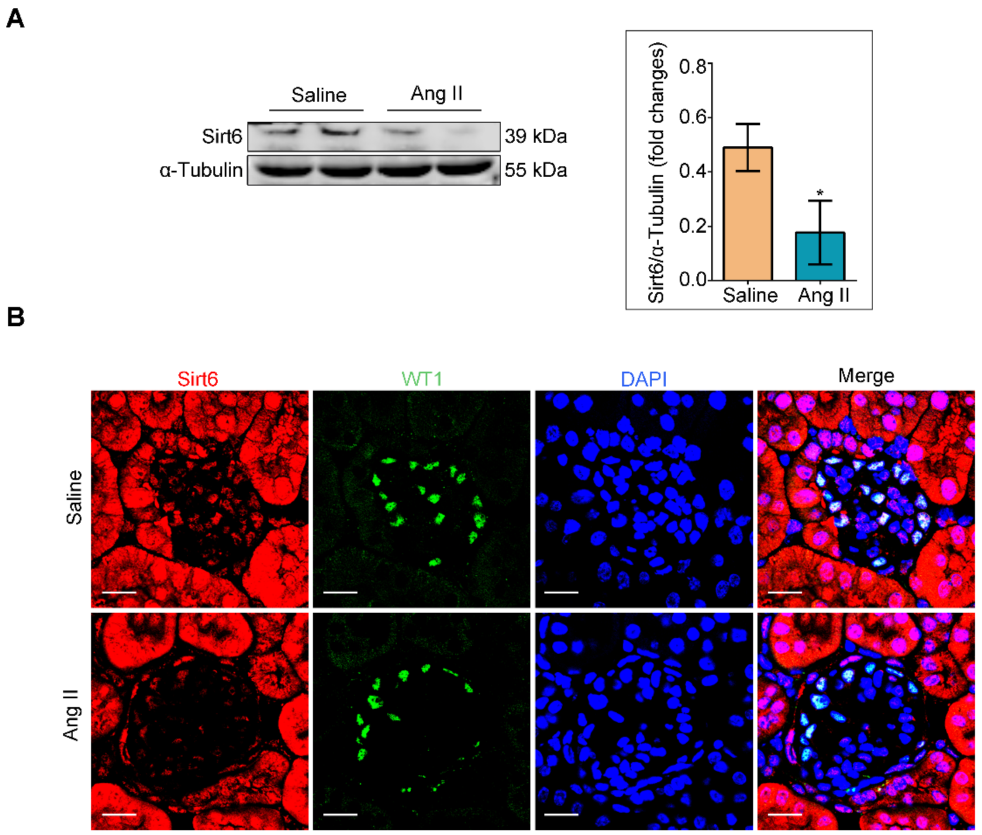

Figure S1. Ang II induces downregulation of Sirt6 in glomeruli and podocytes in vivo. (A) Representative Western blots of Sirt6 and quantification of Sirt6 protein level in glomeruli of each group. **P* < 0.05 versus Saline, n=6. (B) Immunofluorescent staining for Sirt6 in podocytes. WT1 was used as podocyte marker. Scale bars: 20 μm. Saline: Saline infusion group; Ang II: Ang II infusion group. WT1: Wilms' tumor-1.

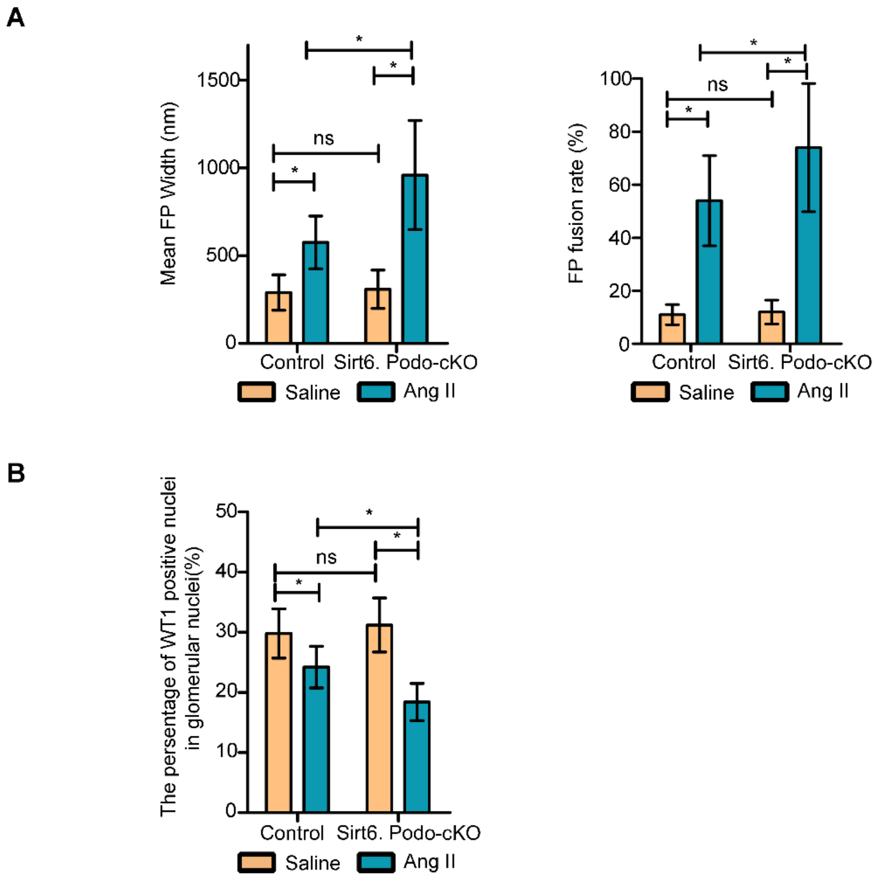

Figure S2. The quantitative analysis of foot process effacement and WT1 positive cells in control and Sirt6.Podo-cKO mice infused with saline or Ang II. (A) Quantitative analysis of foot process effacement in different groups. *$P < 0.05$, n=30.(B) Quantitative analysis of WT1 positive cells in different groups. *$P < 0.05$, n=30. FP: foot process; Control: $Sirt6^{flox/flox}$/Nphs2.Cre- group; Sirt6.Podo-cKO=cKO: Sirt6 podocyte conditional knockout: $Sirt6^{flox/flox}$/$Nphs2.Cre^{+}$ group.

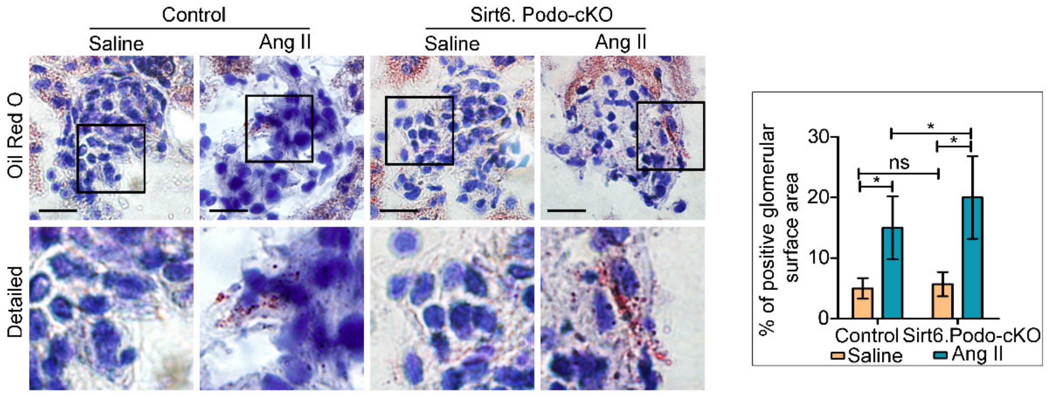

Figure S3. Representative microscopy images and quantification of Oil Red O staining in control and Sirt6.Podo-cKO mice infused with saline or Ang II (original magnification×400). *$P < 0.05$, n=30. Scale bars: 20 μm. Control: Sirt6$^{flox/flox}$/Nphs2.Cre^{-} group; Sirt6.Podo-cKO=cKO: Sirt6 podocyte conditional knockout: Sirt6$^{flox/flox}$/Nphs2.Cre^{+} group.

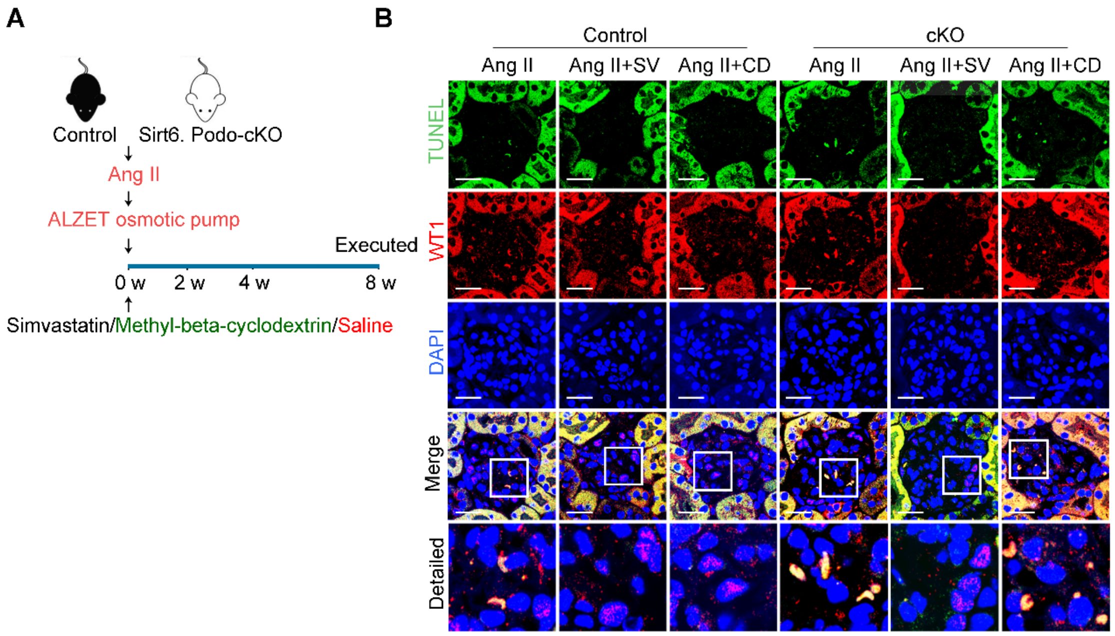

Figure S4. (A) Schematic diagram of the application of cholesterol-lowering agents in the Ang II-induced mouse model. (B) Representative microscopy images of WT1 and TUNEL double staining of kidney sections for each group (original magnification×600). Control: Sirt6$^{flox/flox}$/Nphs2.Cre^{-} group; cKO: Sirt6 podocyte conditional knockout: Sirt6$^{flox/flox}$/Nphs2.Cre^{+} group; Ang II: AngII-infused group; Ang II+SV: Ang II-infused and simvastatin administration group; Ang II+CD: Ang II-infused and CD administration group; CD: cyclodextrin; WT1: Wilms' tumor-1.

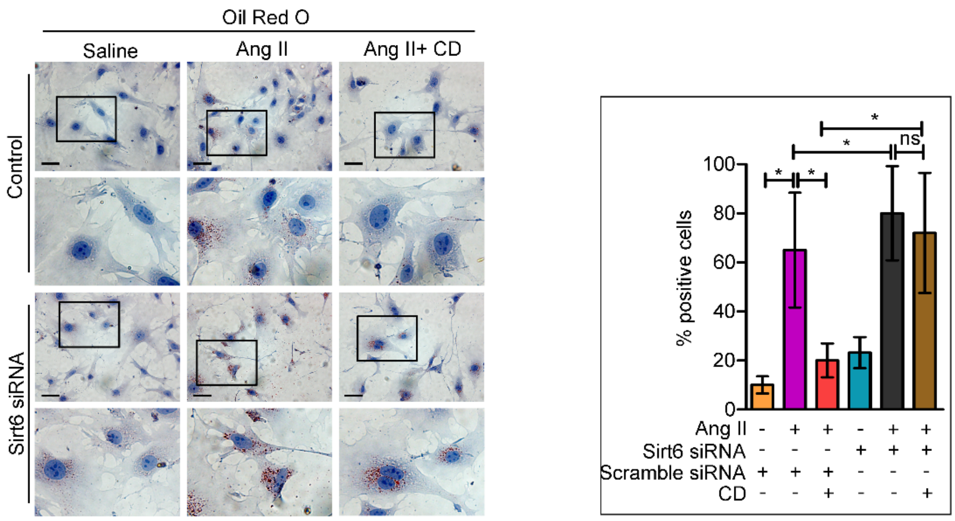

Figure S5. Representative microscopy images and quantification of Oil Red O staining in each group (original magnification×400). Podocytes were transfected with scrambled siRNA, or Sirt6 siRNA before pretreatment with 5 mM CD for 1 h and then stimulated with 10^{-7} M Ang II for 24 h. *$P < 0.05$, n=20. Scale bars: 10 µm. CD: cyclodextrin.
